# Reply to Mathieu et al. Omega 3 Fatty Acids Intake Does Not Decrease the Risk of Rheumatoid Arthritis Occurrence: A Meta-Analysis. Comment on “Tański et al. The Relationship between Fatty Acids and the Development, Course and Treatment of Rheumatoid Arthritis. *Nutrients* 2022, *14*, 1030”

**DOI:** 10.3390/nu15030540

**Published:** 2023-01-20

**Authors:** Natalia Świątoniowska-Lonc, Wojciech Tański, Mateusz Tabin, Beata Jankowska-Polańska

**Affiliations:** 1 Center for Research and Innovation, 4th Military Clinical Hospital with Polyclinic, 50-981 Wroclaw, Poland; 2 Department of Internal Medicine, 4th Military Clinical Hospital with Polyclinic, 50-981 Wroclaw, Poland; 3 Clinical Endocrinology Department, 4th Military Clinical Hospital with Polyclinic, 50-981 Wroclaw, Poland

In response to the comment [1], “Omega 3 fatty acids intake does not decrease the risk of rheumatoid arthritis occurrence: a meta-analysis. Comment on Tanski et al. The Relationship between Fatty Acids and the Development, Course and Treatment of Rheumatoid Arthritis” by Mathieu et al. 2022, first of all, we wanted to congratulate the authors on their interesting meta-analysis. Above all, we would like to highlight the reasons for potential discrepancies between our systematic review [2] and the presented meta-analysis.

First of all, the different design of the article deserves attention. Our systematic review was based particularly on the subjective review method, without any analytical and statistical scope. Therefore, the fact is that we had to treat our summary of findings with caution, and we could not unequivocally exclude or conclude that fatty acids affect the onset, course, and outcome of RA patients. Moreover, the conclusions, which concerned a broad outcome and summarized various nutritional interventions, were summarized as a conceptual model for further verification. It should be emphasized that our study describes the broad impact of a wide range of nutritional interventions, not only omega-3 fatty acids alone, but also the impact of diet and the content of individual fatty acids in the diet of RA patients.

In addition, the qualification criteria based on which the articles have been screened and included in our systematic review, and the meta-analysis by Mathieu et al., are noteworthy to be mentioned. We used different keywords, date range for the literature search, and language of the published papers (English only). In addition, we included only full-text papers for the review.

Considering that the discussed meta-analysis, as a part of the commentary article, without a doubt provides the most reliable, strong, and objective data on the effect of omega-3 fatty acids on the formation of RA. In addition, the meta-analysis will continue to be only one of the few studies presented in the review in which the effect of omega-3 fatty acids on the development of RA is disproven. We emphasize that the substantive scope of our paper is broader than omega-3 acids.
